# Effect of parenting style on the emotional and behavioral problems among Chinese adolescents: the mediating effect of resilience

**DOI:** 10.1186/s12889-024-18167-9

**Published:** 2024-03-13

**Authors:** Jiana Wang, Xinyuan Huang, Zhe Li, Kun Chen, Zhiyu Jin, Jing He, Bingsong Han, Lin Feng, Nana Meng, Cong Yang, Zhuang Liu

**Affiliations:** 1 School of Public Health, Health Science Center, Ningbo University, No.818 Fenghua Road, Ningbo, Zhejiang 315211 People’s Republic of China; 2 Department of Social Medicine, School of Public Health, China Medical University, No. 77 Puhe Road, Shenyang North New Area, Shenyang, Liaoning 110122 People’s Republic of China; 3 Anesthesiology department, The Fourth Affiliated Hospital of China Medical University, , Shenyang, Liaoning, People’s Republic of China; 4 Journal Center of China Medical University, China Medical University, No. 77 Puhe Road, Shenyang North New Area, Shenyang, Liaoning, 110001 People’s Republic of China

**Keywords:** Parenting style, Emotional and behavioral problems, Resilience, Chinese adolescents

## Abstract

**Background:**

Although previous studies have found that parenting style significantly predicts emotional and behavioral problems (EBPs) among Chinese adolescents, the mechanism between different parenting styles and EBPs requires in-depth investigation. In our study, we aimed to investigate the mediating effect of resilience, a positive psychological characteristic, between parenting style and EBPs among Chinese adolescents.

**Methods:**

In this cross-sectional study, we used a multistage stratified cluster random sampling method to collect data in Shenyang, Liaoning Province from November to December 2019. Self-developed questionnaires were distributed to 1028 adolescents aged 10–18. Finally, the study consisted of 895 participants. The bootstrap method was used to investigate the role of resilience as a mediator in the relationship between different parenting styles and EBPs from a positive psychology perspective.

**Results:**

The mean score of EBPs was 12.71 (SD = 5.77). After controlling for variables such as gender, age, left-behind children, family type and family income, resilience partially played a mediating role in the associations of paternal rejection (a × b = 0.051 BCa95%CI:0.023,0.080), maternal rejection (a × b = 0.055 BCa95%CI: 0.024, 0.086), paternal emotional warmth (a × b = -0.139 BCa95%CI: -0.182, -0.099) and maternal emotional warmth (a × b = -0.140 BCa95%CI: -0.182, -0.102), with EBPs. The effect sizes were11.28%, 11.51%, 40.76%, and 38.78%, respectively.

**Conclusions:**

Resilience could partially mediate the relationship between parenting style and EBPs, highlighting that parents should adopt a positive parenting style and that resilience improvement could be effective in reducing EBPs among Chinese adolescents.

## Background

Emotional and behavioral problems (EBPs) are common mental health problems, and an estimated 970 million people in the world were living with mental health problems in 2019 [1]. Adolescence is a transition from childhood to adulthood in which physical and mental health rapidly develop, and the interaction between individual and environmental factors may impact emotional and behavioral problems among individuals aged 10 to 18, leading to a high incidence of mental health problems in adulthood [2–5]. At the same time, mental health problems are major contributing factors to illness and disability, with a remarkable impact on the global burden of disease [6]. The World Health Organization reports that the prevalence of EBPs is approximately 13% among adolescents aged 10–19, including 89 million boys and 77 million girls [6]. According to the Global Burden of Disease study by the Institute for Health Metrics and Evaluation (IHME), depression and anxiety symptoms account for approximately 42.9% of mental disorders, conduct disorders account for 20.1%, and attention-deficit /hyperactivity disorders account for 19.5% [7].

In China, it was estimated that the population of Chinese children and adolescents with EBPs was more than 30 million [8]. Moreover, a meta-analysis revealed that 24.3% of secondary school students in China had depressive disorder, and the pooled incidence gradually increased as they progressed into a higher grade [9]. To promote adolescent mental health, the State Council of China has enacted some plans, including the Healthy China Action Plan (2019–2030) [10] and the Healthy China Action Plan—Children and Adolescents’ Mental Health Plan (2019–2022) [11]. Therefore, the EBPs of Chinese adolescents cannot be ignored, and EBPs during adolescence can have positive lifelong impacts.

The influencing factors that contribute to the risk of EBPs among adolescents have been examined by many researchers, with family factors playing a prominent role [12]. According to Bronfenbrenner’s bioecological theory [13], the family is identified as a microsystem and directly affects children’s emotional responses and behavioral changes. Based on family systems theory, individual health development depends on the wellbeing of the family system, and the parent‒child relationship is an essential subsystem of the family system, in which parenting style is an important component [14]. Parenting style represents the action strategies adopted by parents in the child-rearing process and refers to a series of psychological constructs, generally including emotional warmth, rejection and overprotection [15]. Previous studies have shown that positive and negative parenting styles have different effects on adolescents’ EBPs. Specifically, parenting emotional warmth has a positive effect on emotion and behavior [16]. It is positively correlated with adolescents’ social skills and peer attachment [17, 18]. A study also showed that authoritative parenting can provide emotional support and is related to the healthy adjustment and psychosocial competence of young children [19]. In addition, it has been proposed that an authoritative parenting style can prevent the internalization of symptoms, such as anxiety and depression [20, 21]. Conversely, negative parenting styles, such as less emotional warmth, overprotection or rejection, may be closely related to depression, anxiety, addiction and hostility [22]. Authoritarian parenting limits a child’s autonomy by fostering a strong sense of obedience while offering little emotional responsiveness to their needs [23, 24], and the lack of rules under permissive parenting provides an atmosphere that is insufficient to help the child develop self-control [25]. Thus, authoritarian and permissive parenting are not beneficial to the mental health development of children. Furthermore, maternal and paternal parenting styles also exert different effects on the mental conditions of adolescents, as suggested by recent findings [26]. At the same time, co-parenting, as a unique system in the family, plays an important role in child development. Many studies have shown that differences in parenting attitudes between fathers and mothers determine the problematic behaviors of preschool children [27, 28], and positive co-parenting can help children reduce behavioral problems [29, 30]. Consequently, it is necessary to measure and analyze maternal and paternal parenting styles separately and explore the developmental path of the association between different parenting styles and EBPs to decrease the incidence of EBPs.

Previous studies have focused on EBPs from the perspective of illness and psychopathology, but some researchers have started paying attention to the role of protective factors on adolescents’ EBPs in recent years [31]. Ecosystem theory and developmental contextualism state that the development of individuals depends on the combined influence of external circumstances and individual factors [32–34]. Therefore, this study attempted to explore the influencing factors of EBP among adolescents from a positive development perspective. Resilience, as an important individual’s positive psychological resource, has been increasingly involved in research on adolescent mental health. Research on resilience or psychological resilience is exerting a tremendous fascination on the field of positive psychology, as resilience theory emphasizes strengths rather than vulnerabilities and health development despite adversity [35]. Resilience is defined as a positive personality characterized by the capacity to overcome, steer through and recover from adversity [36]. According to the results of previous studies, resilience is associated with many EBPs among adolescents. Most of these studies on EBPs claim that resilience is a protective factor [37], and those who have higher levels of resilience exhibit fewer EBPs [38]. It is possible that resilience is negatively associated with depression [39], smoking and alcohol use [40]. In addition, ecosystem theory states that there is a close relationship between the external environment and individual characteristics in the process of individual development [32]. Many researchers have suggested that good parenting styles might increase children’s positive psychological reserves and are positively associated with psychological resilience [41]. Studies on the association of resilience and parenting styles have found that children under positive parenting have a high level of resilience [42]. In addition, resilience was considered as a meaningful mediator from previously published studies. According to an investigation, resilience may partially mediate the association between self-harm and suicidal ideation among Chinese left-behind children [43]. In a study on 811 Chinese college students, the author revealed that children who experienced childhood maltreatment tend to have a lower level of resilience, which affects their positive response to childhood maltreatment, leading to an increased risk of aggressive behavior [44]. Thus, resilience can not only effectively reduce the occurrence of children’s problem behaviors but also alleviate the negative impact of risk factors on children’s mental health, which promotes physical and mental health development. However, much remains to be learned about the mechanism between different parenting styles and EBPs from a positive psychology perspective, and whether resilience plays a mediating role in the relationship between different parenting styles and EBPs awaits further investigation. Moreover, most of the existing relevant studies have focused on young adolescents, and only a few reports have been conducted throughout adolescence [45]. In addition, in the Chinese cultural context the situation of parenting style and EBP in adolescents might not be completely consistent with other cultures. Therefore, our study enhanced comprehension of the complex interplay between resilience and parenting style factors in the development of EBP throughout Chinese adolescence, which might provide a new perspective on how parenting styles affect emotional and behavioral problems in the context of Chinese culture and provide some clues for future EBP intervention and improvement of Chinese adolescent mental health.

The purpose of this paper was to understand the association between parenting styles and EBPs and explore the mediating effect of resilience on this association from a positive perspective. Thus, the following two hypotheses in our study were developed: (1) Parenting style and Resilience are significantly associated with EBPs; (2) Resilience plays a mediating role in the relationship between parenting style and EBPs;

## Methods

### Study design and participants

In this cross-sectional study, we used a multistage stratified cluster random sampling method to collect data in Shenyang, Liaoning Province from November to December 2019. We selected two secondary schools and two junior high schools from all public schools in the survey area and ensured that the selected schools included key and ordinary schools. The students were in grades 1 to 3 in junior high school, with an average of 4 classes for each grade, and from grades 7 to 9 in secondary school, with an average of 6 classes for each grade. Overall, 1,028 participants were selected in this study. The questionnaire was self-developed and contained general characteristics, parenting styles, resilience, and EBPs. Before data collection, each participant and their parents/legal guardians signed an informed consent form, which explained the purpose and content of the study. Finally, 1028 students agreed to participate in the study and became our study subjects. After checking for invalid data, such as incomplete questionnaires, missing data, and incorrect data registration, the number of participants decreased to 895 students in our study. The effective response rate was 87.06%. There were no demographic differences among the participants. The sample included male (54.08%) and female (45.92%) students, with ages ranging from 10 to 18 years (mean = 15.38, SD = 1.41).

### Ethics statement

Statement that the study was performed in accordance with relevant guidelines and regulations. This research was approved by the Ethics Committee on Human Experimentation of China Medical University [CMU-20180228057]. Before the beginning of the data collection, each participant and their parents/legal guardians signed an informed consent form, which explained the purpose and content of the study and had the right to opt out of the study at any time. In this study, participation was completely anonymous, confidential and voluntary.

### Measures

#### Measurement of EBPs

The EBPs were estimated using the self-reported version of the 25-item Strengths and Difficulties Questionnaire (SDQ) devised by Goodman [46], which has been widely used to observe and evaluate adolescents’ self-perception of emotional and behavioral problems [47, 48]. It includes 5 dimensions (emotional symptoms, conduct problems, hyperactivity, peer problems and prosocial behavior). The response for each item was scored on a 3-point Likert scale: 0 = not true, 1 = true, 2 = completely true. The prosocial behavior factor is the strength factor, with a higher score indicating greater prosocial behavior, which differs from the first four difficulty factors. The total difficulty score is the sum of the first four factors, ranging from 0 to 40. The total difficulty score was used to assess EBP among Chinese adolescents, with a higher score indicating more problems in this study. According to the norm set by the Shanghai Mental Health Center of China [49], the total difficulties score was categorized as normal (0–13 points), borderline (14–16 points), and abnormal (17–40 points). The existing research shows that the Chinese version of the SDQ has been widely used among children and adolescents in China with good reliability and validity [50–52]. The Cronbach’s alpha was 0.79 in this study.

#### Measurement of parenting styles

The paternal and maternal parenting styles were estimated with a Short-Form of the Egna Minnen Barndoms Uppfostran (S-EMBU) according to the purpose of the study [53]. The scale can measure paternal and maternal parenting styles separately. This 23-item scale includes 3 dimensions (rejection, overprotection and emotional warmth), all of which have 4-point responses: “1 = never”, “2 = occasionally”, “3 = often”, and “4-very often”. A higher combined score indicates a parenting style that was adopted more frequently. The Cronbach’ s alpha coefficients of the paternal and maternal parenting styles subscales were 0.82 and 0.78, respectively.

#### Measurement of resilience

Resilience was assessed with the Chinese version of the Connor-Davidson Resilience Scale (CD-RIS-C) [54]. Jianxin Zhang et al. divided the 25-item scale into three dimensions (tenacity, strength, and optimism), all of which have a 5-point response: “0 = not true at all”, “1 = rarely true”, “2 = sometimes true”, “3 = often true”, and “4 = true nearly all of the time” [54]. The total score ranges from 0–100, and a higher score on the CD-RIS-C represents greater resilience. The Chinese version of the Connor-Davidson Resilience Scale (CD-RIS-C) has been widely used in Chinese adolescents, and it has adequate reliability and validity [55, 56]. In this study, the Cronbach’s alpha was 0.936, each dimension was 0.967, 0.814 and 0.727, respectively, and the split-half reliability coefficients was 0.879. The confirmatory factor analysis (CFA) showed that the 3-factor model fits well (X^2^/df = 4.097, RMSEA = 0.059, CFI = 0.929, TLI = 0.913, IFI = 0.929, GFI = 0.915, NFI = 0.908).

#### Sociodemographic characteristics

Sociodemographic characteristics include gender, age (yrs)**,** left-behind children**,** family type and family monthly income (RMB, yuan)**.** Age was categorized as ≤ 15 and > 15. The left-behind children were defined as yes or no (left-behind children represent children under 16 who have remained in rural regions of China for more than 6 months, with at least one of their parents moving to other cities for work)**.** Family type was categorized as nuclear family and single/other family. Family monthly income was categorized as ≤ 3000 yuan and > 3000 yuan (according to average income in this area).

### Statistical analysis

Statistical analysis was conducted with IBM SPSS for Windows (Ver. 24.0). We used Pearson’s chi-square (χ^2^) to describe the group differences in total difficulties. The correlations among quantitative variables were measured using Pearson’s correlation. Third, we used the PROCESS macro of SPSS developed by Preacher and Hayes to conduct the analysis of the mediation effect with a bootstrap approach. After controlling for sociodemographic characteristics in the regression analysis, parenting styles were fitted as predictor variables, with EBPs as an outcome variable and resilience as mediator. The first step was to test the total effect of parenting styles on EBPs (c path), and the second step was to estimate the mediation effect of resilience (c’ path), representing the direct effect of parenting styles on EBPs through resilience. The possibility of mediation was speculated when the mediation effect (c’ path coefficient) was smaller than the total effect (c path coefficient) or was not significant. The significance of the indirect effect (a*b = c-c’) was speculated by a bias-corrected and accelerated 95% confidence intervals (BCa 95% CIs), and there was a significant mediation when BCa 95% CIs did not include 0. Statistical significance was considered as* p* < 0.05 in this study.

## Results

### Sociodemographic characteristics of the participants

The results regarding the sociodemographic characteristics of the participants and comparisons of the total difficulties of the SDQ are presented in Table 1. There were 281 Chinese children’s difficulty scores were borderline/abnormal, with an estimated incidence of 31.40%. There were significant differences in the distribution of difficulty scores among Chinese children in terms of sex, age, left-behind children and family type. Compared with girls, boys were more likely to suffer from abnormal problems (*p* < 0.05). Children over15 years old had more abnormal problems than those aged 15 or younger (*p* < 0.05). Left-behind children had more abnormal problems than the ordinary children (*p* < 0.05). Children living in small/large families had fewer abnormal problems (*p* < 0.05) and the distribution of difficulty scores was not significantly different by family income.

**Table 1:** Sociodemographic characteristics and comparisons of total difficulties (*n* = 895)

Sociodemographic Variables	N (%)	Total difficulties			*X*^*2*^
		**Normal(*n* = 614)**	**Borderline(*n* = 204)**	**Abnormal(*n* = 77)**	
**Gender**					
Boy	411(45.92)	260(63.26)	106(25.79)	45(10.95)	11.019**
Girl	484(54.08)	354(73.14)	98(20.25)	32(6.61)	
**Age**					
≤ 15	313(35.00)	239(76.36)	56(17.89)	18(5.75)	13.846***
> 15	582(65.00)	375(64.43)	148(25.43)	59(10.14)	
**Left-behind children**					
Yes	137(15.31)	80(58.39)	40(29.20)	17(12.41)	8.091*
No	758(84.69)	534(70.45)	164(21.64)	60(7.91)	
**Family type**					
Small family or Big family	675(75.42)	482(71.41)	144(21.33)	49(7.26)	11.480**
Single family or other	220(24.58)	132(60.00)	60(27.27)	28(12.73)	
**Family income**					0.500
≤ 3000	380(42.46)	261(68.68)	89(23.42)	30(7.90)	
> 3000	515(57.54)	353(68.54)	115(22.33)	47(9.13)	

^*^P < *0.05 Statistically significant at confidence level of 95%*

^**^P < *0.01 Statistically significant at confidence level of 99%*

^***^P < *0.001 Statistically significant at confidence level of 99.9%*

### Correlation between study variables

The relevant results for this section are given in Table 2. Correlations among quantitative variables were measured using Pearson’s correlation. The mean score of total difficulties was 12.71 (SD = 5.77). The results showed that rejection and emotional warmth both from fathers and mothers were significantly correlated with EBPs, as well as resilience (*p* < 0.001). In particular, the paternal and maternal emotional warmth were positively correlated with resilience, but paternal and maternal rejection were negatively correlated with resilience. However, there were no significant correlations between parental overprotection and resilience (*p* > 0.05). In addition, parental overprotection was significantly correlated with EBPs (*p* < 0.001).

**Table 2:** Correlations between parenting styles, resilience, and EBPs

Variables	1	2	3	4	5	6	7	8
1.FR	1							
2.MR	0.851 ***	1						
3.FEW	-0.155 ***	-0.186 ***	1					
4.MEW	-0.243 ***	-0.247 ***	0.808 ***	1				
5.FO	0.666 ***	0.568 ***	0.203 ***	0.080 *	1			
6.MO	0.470 ***	0.580 ***	0.109 ***	0.149 ***	0.745 ***	1		
7.Resilience	-0.138 ***	-0.142 ***	0.390 ***	0.385 ***	0.060	0.057	1	
8.TDS	0.394 ***	0.399 ***	-0.291***	-0.299 ***	0.198 ***	0.163***	-0.371***	1
M	13.65	13.79	19.99	20.62	17.51	17.94	59.35	12.71
SD	4.92	4.72	4.80	4.63	4.07	3.91	16.80	5.77

*FR* Father Rejection, *MR* Mother Rejection, *FEW* Father Emotional warmth, *MEW* Mather Emotional Warmth, *FO* Father Overprotection, *MO* Mother Overprotection, *TDS* Total Difficulty Score

^*^P < *0.05 Statistically significant at confidence level of 95%*

^**^P < *0.01 Statistically significant at confidence level of 99%*

^***^P < *0.001 Statistically significant at confidence level of 99.9%*

### Mediation analysis

After finding internal links among parenting styles, resilience, and EBPs, this study examined the potential mediating role of resilience between different parenting styles and EBPs. To achieve detailed insight into different parenting styles, the effect of resilience was explored between the six subscales of parenting style and EBPs by bootstrapping.

The results of the regression analysis of different parenting styles on resilience and EBPs among Chinese children can be seen in Table 3. After controlling for variables, such as gender, age, left-behind children, family type and family income, the results showed that rejection from fathers and mothers significantly positively predicted EBPs (*β* = 0.452, *p* < 0.001; *β* = 0.478,* p* < 0.001) and significantly negatively predicted resilience (*β* = -0.473, *p* < 0.001; *β* = -0.507, *p* < 0.001). Emotional warmth from fathers and mothers significantly negatively predicted EBPs (*β* = -0.341, *p* < 0.001; *β* = -0.361, *p* < 0.001) and significantly positively predicted resilience (*β* = 1.366, *p* < 0.001; *β* = 1.389, *p* < 0.001). Father overprotection and mother overprotection significantly positively predicted EBPs (*β* = 0.272, *p* < 0.001; *β* = 0.234, *p* < 0.001). However, paternal and maternal overprotection did not significantly predict resilience. When resilience was added, the absolute value of rejection *β* and emotional warmth *β* both fathers and mothers was diminished. Thus, resilience could serve as mediator in the association between the four subscales of parenting styles (Father rejection, Father emotional warmth, Mother rejection and Mother emotional warmth) and EBPs. The total, direct and indirect effects are presented in Table 4.

**Table 3:** Regression analysis of parenting styles on resilience and EBPs in Chinese adolescents

**Regression Equation**		**Model Fit**			**Significance**	
**Outcome Variables**	**Predictor Variables**	***R***^***2***^	***F***		***Β***	***T***
EBP	Father rejection	0.215	40.624***	*c*_*1*_	0.452	12.793***
Resilience	Father rejection	0.044	6.777***	*a*_*1*_	-0.473	-4.166***
EBP	Father rejection	0.310	56.911***	*c’*_*1*_	0.401	11.975***
	Resilience			*b*_*1*_	-0.108	-11.025***
EBP	Mother rejection	0.219	41.370***	*c*_*2*_	0.478	12.954***
Resilience	Mother rejection	0.045	6.932***	*a*_*2*_	-0.507	-4.274***
EBP	Mother rejection	0.312	57.447***	*c’*_*2*_	0.423	12.100***
	Resilience			*b*_*2*_	-0.107	-10.977***
EBP	Father emotional warmth	0.149	25.9 67***	*c*_*3*_	-0.341	-9.052***
Resilience	Father emotional warmth	0.174	31.104***	*a*_*3*_	1.366	12.636***
EBP	Father emotional warmth	0.222	36.100***	*c’*_*3*_	-0.202	-5.160***
	Resilience			*b*_*3*_	-0.102	-9.088***
EBP	Mother emotional warmth	0.154	26.868***	*c*_*4*_	-0.361	-9.325***
Resilience	Mother emotional warmth	0.170	30.282***	*a*_*4*_	1.389	12.444***
EBP	Mother emotional warmth	0.225	36.760***	*c’*_*4*_	-0.221	-5.508***
	Resilience			*b*_*4*_	-0.101	-9.028***
EBP	Father overprotection	0.107	17.772***	*c*_*5*_	0.272	6.020***
Resilience	Father overprotection	0.029	4.356***	*a*_*5*_	0.245	1.787
EBP	Father overprotection	0.244	40.805***	*c’*_*5*_	0.303	7.282***
	Resilience			*b*_*5*_	-0.129	-12.646***
EBP	Mother overprotection	0.096	15.623***	*c*_*6*_	0.234	4.925***
Resilience	Mother overprotection	0.028	4.304***	*a*_*6*_	0.244	1.700
EBP	Mother overprotection	0.230	37.847***	*c’*_*6*_	0.265	6.036***
	Resilience			*b6*	-0.128	-12.448***

*EBP* Emotional and behavioral problems

^*^P < *0.05 Statistically significant at confidence level of 95%*

^**^P < *0.01 Statistically significant at confidence level of 99%*

^***^P < *0.001 Statistically significant at confidence level of 99.9%*

**Table 4:** Total, indirect, direct effects, effect size

Types of Parenting styles		Estimate	Boot SE	Boot CI LL	Boot CI UL	Effect Size
Father rejection	Total effect	0.452	0.035	0.383	0.522	
	Indirect effect	0.051	0.014	0.023	0.080	11.28%
	Direct effect	0.401	0.034	0.335	0.467	
Mother rejection	Total effect	0.478	0.037	0.405	0.550	
	Indirect effect	0.055	0.016	0.024	0.086	11.51%
	Direct effect	0.423	0.035	0.355	0.492	
Father emotional warmth	Total effect	-0.341	0.038	-0.415	-0.267	
	Indirect effect	-0.139	0.021	-0.182	-0.099	40.76%
	Direct effect	-0.202	0.039	-0.279	-0.125	
Mother emotional warmth	Total effect	-0.361	0.039	-0.437	-0.285	
	Indirect effect	-0.140	0.021	-0.182	-0.102	38.78%
	Direct effect	-0.221	0.040	-0.300	-0.142	

*SE* standard error, *CI* confidence intervals, *LL* lower limit, *UL* upper limit

**Fig. 1:**
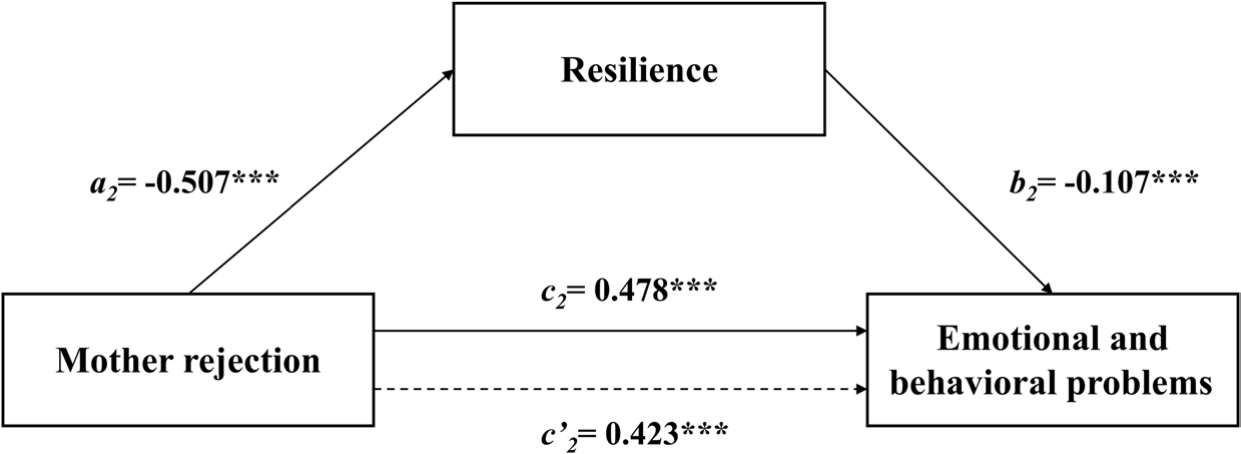
A path model for mediation via resilience in the association between father rejection and EBPs. Note: ***P < 0.001

**Fig. 2:**
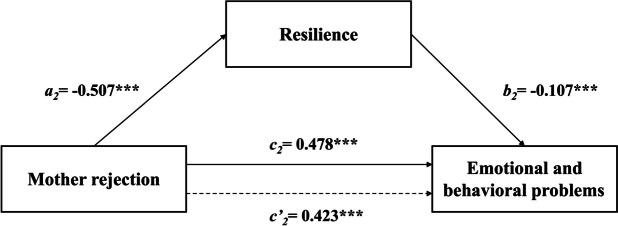
A path model for mediation via resilience in the association between mother rejection and EBPs. Note: ***P < 0.001

**Fig. 3:**
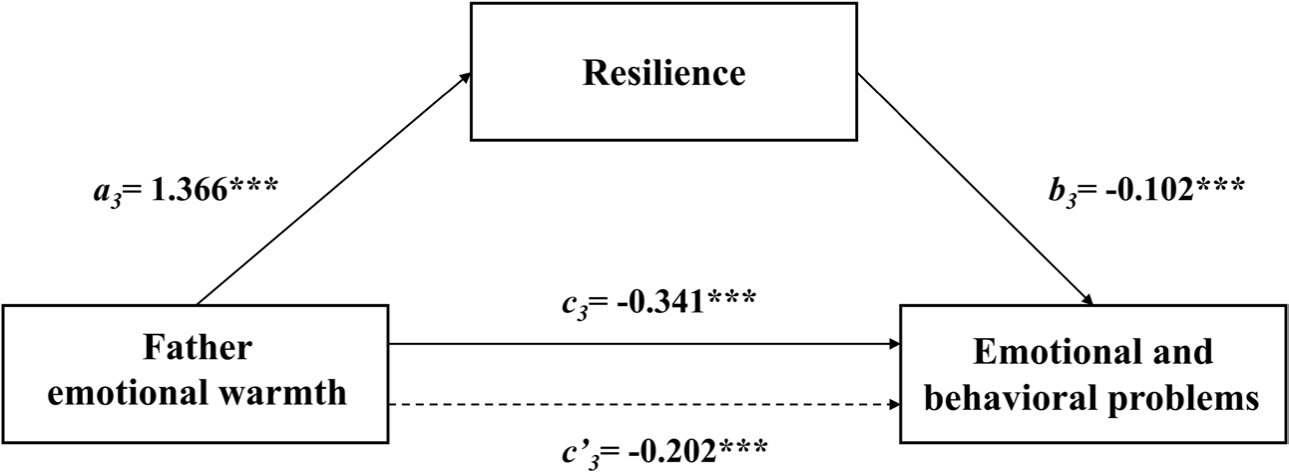
A path model for mediation via resilience in the association between father emotional warmth and EBPs. Note: ***P < 0.001

**Fig. 4:**
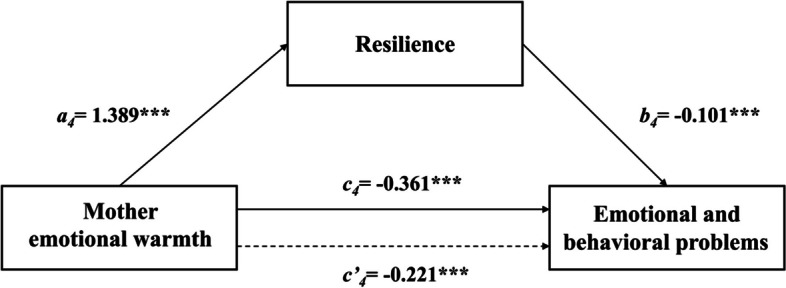
A path model for mediation via resilience in the association between mother emotional warmth and EBPs. Note: ***P < 0.001

The mediation effect analysis showed a bootstrap 95% confidence interval excluding 0 for four subscales of parenting styles (Father rejection, Mother rejection, Father emotional warmth, and father emotional warmth), indicating that resilience partially mediated the associations of father rejection, mother rejection, father emotional warmth, and mother emotional warmth with EBPs. The effect sizes were 11.28%, 11.51%, 40.76%, and 38.78%, respectively. The path coefficients of the mediator model are shown in Figs. 1, 2, 3 and 4.

## Discussion

The study aimed to investigate the association between different parenting styles and EBPs, as well as the mediating effect of resilience on this specific association among Chinese adolescents in Shenyang, Liaoning Province. Although previous studies have found that parenting styles significantly predict EBPs, most researchers have explored this specific association from the perspective of illness and psychopathology. In addition, the mechanism between different parenting styles and EBPs awaits further investigation from a positive perspective. Accordingly, the results of this study are critical for understanding the mediating effect of resilience, a positive psychological factor, on the relationships between different parenting styles and EBPs. The meaningful results and implications of this study are summarized as follows.

The results show that the mean score of EBPs was 12.71 ± 5.77 among Chinese adolescents in the city of Shenyang, Liaoning Province. According to the total difficulty score, the detection rate of abnormal EBPs in adolescents was 8.6%. This differs from a previous study [57], which showed that the detection rate of abnormal EBPs was 11.2% in Hunan Province. In addition, the present study confirms that boys may suffer from mental disorders compared with girls. The findings in this study are consistent with the previous findings that boys exhibited more mental health problems (13.8%) than girls (12.5%) in the same age group [58].

In this study, our findings indicate that parenting style was substantially correlated with EBPs among Chinese adolescents, which was in line with other studies [59, 60]. According to our results, parental emotional warmth was negatively associated with the EBPs and subscales. In contrast, rejection and overprotection were positively associated with EBPs. It is noted that positive parenting may promote individuals’ development of coping mechanisms, such as optimistic emotions, adaptive justifications, and sufficient social support, which help them avoid EBPs. Silk JS et al. indicated that emotional warmth promotes emotional regulation and stress coping strategies, which reduce the risk of behavioral problems [61]. Similarly, a study confirmed that a negative parenting style aggravates emotional problems and forms an unsafe environment in which the negative emotions persist [62]. Additionally, positive parenting could decrease adolescents’ misbehavior, perhaps in part because parental support and warmth facilitate parent–child communication so that parents understand information about their children’s activities and exert appropriate behavioral control [63]. In simpler terms, adolescents who are given more emotional warmth from their family may exhibit fewer EBPs. Thus, parents should adopt positive parenting to decrease adolescents’ EBPs.

Resilience is significantly associated with EBPs in this study. Our findings demonstrate that adolescents with a high level of resilience are likely to exhibit fewer EBPs. Previous research has confirmed that individuals who are resilient exhibit fewer symptoms of depression [57], academic difficulties [64], and social difficulties [65] in the crucial period of rapid development in body and mind. These results have also been found by other similar studies [66, 67], which underline the importance of improving the level of resilience. Adolescents with lower resilience tend to experience negative emotions for a longer time, giving rise to a variety of psychological problems because they cannot adopt reasonable responses to adjust themselves. It is possible for adolescents with a high level of resilience to effectively regulate their negative feelings and resolve conflicts or contradictions in the face of difficulties. A study indicated that resilience affects the mental health of individuals through three mechanisms (recovery mechanism, protection mechanism, and promotion mechanism) [68]. Therefore, resilience could play a crucial role in reducing adolescent EBPs.

In addition, parenting style is an important influencing factor for resilience, and it can predict the level of resilience. In this study, we found that emotional warmth positively predicted resilience, whereas rejection negatively predicted resilience. In other words, adolescents who have experienced positive parentings tend to have a high level of resilience to overcome frustrations and cope with adversity. These adolescents develop self-confidence and self-efficacy as a foundation for high resilience [69]. Emotional warmth can promote the development of positive psychological qualities. In contrast, adolescents under negative parenting styles are inclined to have a weak ability to repair themselves in the face of difficulties or dangers. Moreover, our study found that the correlation between overprotection and resilience was not significant. This result was consistent with a study that found that there was no prominent correlation between a permissive parenting style and resilience [66]. Therefore, parents should give their children more emotional warmth and build a good parent‒child relationship to improve adolescents’ psychological resilience.

The PROCESS results showed that emotional warmth and rejection predicted EBPs via resilience. Resilience partially mediated the associations of father rejection, father emotional warmth, mother rejection and mother emotional warmth, with EBPs in adolescents, while there was no mediating effect of resilience between parental overprotection and EBPs. The effect sizes of these associations were 11.28%, 11.51%, 40.76%, and 38.78%, respectively. Compared with maternal emotional warmth, resilience has the strongest mediating effect between paternal emotional warmth and EBPs, which may be because the father has played a specific role in promoting resilience; that is, paternal emotional support tends to have a greater effect on resilience than other protective factors [70, 71]. One study has shown that if paternal emotional warmth is provided, children can exhibit a higher level of mental health [72]. The earliest studies suggested that a supportive or warm parenting style during a child’s formative stage significantly decreases the risk of psychological disorders in the future, as it promotes the development of resilience [73]. This positive association between positive parenting, high resilience, and health mental condition might be considered a protective influence of parenting style [74]. On this basis, the main conclusion is that resilience could be identified as a prominent mediator in the association between parenting styles and EBPs.

### Limitations

There are several limitations in the current study to be noted while reviewing our findings. One limitation is that the results are based on a cross-sectional study that did not incorporate multiple time points; thus, the relationships among parenting style, resilience, and EBPs cannot be determined. The results and conclusions drawn from this study need to be confirmed by further long-term longitudinal studies. Second, information on parenting styles, resilience, and behavioral problems was collected from adolescent-reported data and collected at the same time. This inevitably has the potential to be introduce recall, response and common method bias regarding the artificial correlation due to similarity in measurement methods, which may affect the accuracy and completeness of the collected data. Even so, some researchers believe that the adolescent is one of the most reliable sources for evaluating parenting style because it is less conditional by social desirability [42].

### Implications for research and practice

Emotional and behavioral problems (EBPs) are public health problems, and importance must be attached by family, scholars and administration. This research focused on the mediating effect of resilience on the association between parenting style and EBPs among Chinese adolescents, providing significant theoretical and practical implications for understanding the EBPs of Chinese adolescents. First, the results have enriched the ecological framework theory, family system theory, and the resilience model, which revealed the influence of family environment and positive psychological characteristics on adolescent development but also help to understand the relationship between different parenting styles and mental health problems from the perspective of resilience. Second, from an academic point of view, we suggest that parents adopt more active parenting styles, consider problems from the perspective of their children, and care for the mental conditions of children during the rapid development of their mind and body. At the same time, in the process of education, education workers should strengthen students’ frustration education and guide them to overcome setbacks to improve their resilience. Third, the longitudinal studies should be designed by researchers to discover the causal relationships of adolescents in the future.

## Conclusions

In this cross-sectional study, it is concluded that emotional warmth was negatively associated with EBPs, but rejection or overprotection was positively associated with EBPs. In addition, resilience plays a prominent mediating role in the association of parental emotional warmth and EBPs, as well as parental rejection and EBPs, indicating that positive parenting may reduce the EBPs of Chinese adolescents by improving the resilience of adolescents but negative parenting against the development of resilience, in turn causing more EBPs. The results of the study offer new perspectives that parents should adopt positive parenting and resilience improvement could be effective in reducing the risk of EBPs among Chinese adolescents.

## Data Availability

The datasets analyzed during the current study are available from the corresponding author on reasonable request.

## Abbreviations

EBPs: Emotional and behavioral problems
SD: Standard deviation
BCa95%CI: Bias-corrected and accelerated 95% confidence interval
FR: Father Rejection
MR: Mother Rejection
FEW: Father Emotional warmth
MEW: Mather Emotional Warmth
FO: Father Overprotection
MO: Mother Overprotection
TDS: Total Difficulty Score
SDQ: Strengths and Difficulties Questionnaire
S-EMBU: Short-Form of the Egna Minnen Barndoms Uppfostran
CD-RIS-C: Connor-Davidson Resilience Scale
